# Can We Cryopreserve the Sperm of COVID-19 Patients During the Pandemic?

**DOI:** 10.3389/fendo.2022.753267

**Published:** 2022-05-30

**Authors:** Yongming Wu, Xiaoxue Zhang, Zhiqiang Wang, Xinyi Xia

**Affiliations:** ^1^ Human sperm bank, the First Affiliated Hospital of Guangxi Medical University, Nanning, China; ^2^ COVID‐19 Research Center, Institute of Laboratory Medicine, Jinling Hospital, Nanjing University School of Medicine, The First School of Clinical Medicine, Southern Medical University, Nanjing, Jiangsu, China; ^3^ Jiangsu Province Key Laboratory for Molecular and Medical Biotechnology, College of Life Science, Nanjing Normal University, Nanjing, China

**Keywords:** COVID-19, SARS-CoV-2, semen, cryobanks, cryopreservation

## Abstract

An extreme strain has been placed on healthcare facilities in the COVID-19 era. Initial stage of the pandemic, national and international societies for reproductive medicine suggested the suspension of new IVF treatments and non-essential cryopreservation of gametes. Accordingly, the demands of cryopreservation of semen with COVID-19 patients also was suspended by some of cryobanks to protect staff and patients from unnecessary viral exposure at the acute stage. However, the pandemic may stay with us longer than expected. In addition, there will be some male COVID-19 patients with cancer or critically illness who needs to cryopreserve their semen before medical treatments, otherwise they might loss the chance of getting their own offspring. In this document, we summarize available evidence to deepen and expand awareness of feasibility of sperm cryopreservation and propose some suggestions to help cryobanks carry out sperm preservation procedure for COVID-19 male patients.

## Introduction

Up to the end of June, 2021, the outbreak of Corona Virus Disease 2019(COVID-19), which has lasted for more than for a year and a half, is so prevalent around the world. Although epidemiologists’ forecasts and timelines vary, they all agree on COVID-19 is here to stay, and the future depends on a lot of unknown (1). At present, national measures to reduce person to person transmission have succeeded in de‐escalating of COVID‐19 pandemic crisis, but the development of the epidemic in most countries is still far from optimistic. Globally, infections with SARS-CoV-2 virus are continuously rising with mounting numbers of deaths. At the time of writing, more than 160 million confirmed COVID-19 cases and over 4.0 million confirmed deaths have been reported (2). SARS-CoV-2 mainly affects the lungs, but emerging evidence suggests that the virus is also capable of infecting other organs, such as heart, kidney and human reproductive organs.

After the World Health Organization announced the onset of the SARS-CoV-2 pandemic, several fertility societies worldwide responded by recommending that fertility clinics should suspend the new IVF treatment, for patients who have the demands of fertility preservation, freezing gametes is recommended (3, 4). With the accumulation of data and experience, cryobanks have re-opened step wisely, but their activities, including semen cryopreservation, still be restricted to some extent. Some of sperm cryobanks only accept asymptomatic patients who are about to undergo radio- and/or chemotherapy while the COVID-19 patients who are keen to access fertility preservation were curbed. Because we still can’t answer following questions (with incomplete data) as yet: (1) Whether SARS-CoV-2 is present in the semen of COVID-19 patients? (2) Can the strategies of mitigating SARS-CoV-2 cross- contamination risk be established at cryopreservation stage. (3) Can SARS-CoV-2 from frozen semen be eliminated effectively by repeated washing to lessen infectivity?

The prudent measures may be the safest strategy at the stage to minimize the risks related to SARS-CoV-2 during the pandemic. However, over one year of inactivity, an inevitable issue is a backlog of COVID-19 patients with cancer or critically illness. Notably, compared with women, men are more vulnerable to infection in the outbreak, especially those of reproductive age, and their mortality of COVID-19 is also higher (5–8). Therefore, it is a significant subject for specialists to assess the necessity of semen cryopreservation while also developing safe and effective measures to meet the fertility preservation demands of COVID-19 patients.

## The Effect of SARS-CoV-2 on Male Reproductive Function

Emerging evidence indicate that the changes of pathological structure and proteomics in the testicular tissue (9–11), disorders of sex hormones (12–15), damage to spermatogenesis (16, 17), and decreased sperm quality of COVID-19 patients (12, 18). These studies mentioned above suggest that SARS-CoV-2 can adversely affect multiple reproductive organs at least in a short term. The main mechanisms of SARS-CoV-2 impacting male fertility potential can be summarized as follows: (a) SARS-CoV-2 may lead to impairment of the blood–testis barrier and attack the germ cells directly (19); (b) SARS-CoV-2 affect the activity of the hypothalamic–pituitary–testicular (HPT) axis and lead to dysfunction in release of reproductive endocrine hormones (20); (c) Possible inflammatory responses and oxidative stress induced by SARS-CoV-2 disrupt the reproductive system (21); (d) the fever caused by infection interferes with normal reproductive physiology (22). Of note, the mechanisms mentioned above usually coexist and have a synergistic effect on impairing male reproductive function (23).

## Possibility of SARS-CoV-2 Virus in Semen, EPS and Testis

To date, more than 27 viruses (HIV, mumps, zika, among others) have been found in semen. Some may be particularly persistent, like the Zika virus detected in the semen of asymptomatic men for up to 1 year after healing (24). Researchers also try to determine whether SARS-CoV-2 is present in semen of COVID-19 patients. The conclusion provides an especially important reference basis for sexual partner, semen processing, sperm cryopreservation and assisted reproductive technology (ART). For cryobanks, if there is active SARS-CoV-2 in semen, high attention should be paid to semen analysis and sperm preservation, as staff would be at great risk of infection. Fortunately, although there is still controversy concerning the presence of SARS-CoV-2 in human semen, available data increasingly appears to indicate the absence of SARS CoV-2 in semen (**Table 1**). Conversely, few studies have shown that viral RNA can be detected in semen from SARS-CoV-2 positive patients (18, 34). Considering that the distal urinary and reproductive tracts overlap in males, the RNA detected in semen may be just a residual of urinary shedding, which could lead to false-positive results. Thus, from the little data available, the risk that SARS-CoV-2 might be transmitted through semen seems fairly low in COVID-19 patients. What to need to be pointed out is, these studies used small sample sizes and examined confirmed cases of COVID‐19 during recovery, the possibility that SARS-CoV-2 is present in semen cannot be completely ruled out. Taking the factor into account, large-scale and multi-center studies are needed to draw convincing conclusions about the presence of SARS-CoV-2 in semen.

**Table 1:** Summary of detection of SARS-CoV-2 in the male reproduction system.

Number Study		Sample size(n)	Stage of Disease	Reported positive results of SARS-CoV-2 detection			
				Testis biopsies	Semen	Prostate/EPS	Other samples
*1*	(Guo, Zhao et al., 2021) (25)	23	recovered:11 recent infection:12	ND	0/23	ND	ND
2	(Holtmann, Edimiris et al., 2020) (26)	20	recovered men:18; acute stage:2	ND	0/20	ND	ND
3	(Li, Xiao et al., 2020) (17)	29	recovered:23 deceased:6	ND	0/23	ND	ND
4	(Ma, Xie et al., 2021) (12)	12	recovered men:11; Treatment stage:1	ND	0/12	ND	ND
5	(Pan, Xiao et al., 2020) (27)	34	recovered:34	ND	0/34	ND	ND
6	(Kayaaslan, Korukluoglu et al., 2020) (28)	16	Acute Stage	ND	0/16	ND	ND
7	(Ruan, Hu et al., 2021) (29)	74	recovered	ND	0/70	0/61	0/74 (urine)
8	(Rawlings, Ignacio et al., 2020) (30)	6	recovered	ND	0/6	ND	ND
9	(Burke, Skytte et al., 2021) (31)	18	Symptomatic:15 Asymptomatic:3	ND	0/18	ND	ND
10	(Paoli, Pallotti et al., 2020) (32)	1	recovered	ND	0/1	ND	0/1 (urine)
11	(Zhang, Wang et al., 2020) (33)	10	positive nasal swab for SARS-CoV-2:3 nasal swab for SARS-CoV-2 turned negative:7	ND	ND	0/10	ND
12	(Li, Jin et al., 2020) (34)	38	recovered: 23 acute stage :15	ND	6/38	ND	ND
13	(Gacci, Coppi et al., 2021) (18)	43	recovered	ND	1/43	ND	2/43 (urine)
14	(Machado, Barcelos Barra et al., 2021) (35)	15	no symptoms: 2 mild symptoms:13	ND	1/15	ND	ND
15	(Temiz MZ et al., 2021) (36)	20	before treatment:10 after treatment:10	ND	0/20	ND	ND
16	(Song, Wang et al., 2020) (37)	13	recovered:12 deceased:1	0/1	0/12	ND	ND
17	(Yang, Chen et al., 2020) (10)	12	deceased	0/12	ND	ND	ND
18	(Bian and Team 2020) (38)	NR	deceased	+ (NR)	ND	ND	ND
19	(Achua, Chu et al., 2021) (39)	6	deceased	1/6	ND	ND	ND
20	(Ma, Guan et al., 2021) (40)	5	deceased	2/5	ND	ND	ND

ND, not determined indicates that assays were not performed; NR, no specific data reported; +, results were positive.

ACE2 and transmembrane serine protease 2 (TMPRSS2) are highly expressed by the epithelium of the human prostate. Thus, it is possible that SARS-CoV-2 may be affect the prostate and the virus could infiltrate into the prostatic secretion. As an essential component of semen, prostatic secretion is secreted by the prostate which can be collected by prostatic massage. In this review, there are two studies on the EPS, with a total of 71 samples (29, 33). However, according to these research results, SARS-CoV-2 RNA were not detected in all EPS samples. These results indicate that the virus may not exist in EPS and further supports that there is little possibility of SARS-CoV-2 in the semen of COVID-19 patients.

Due to expressing the ACE2 receptor, a target for SARS-CoV-2 infection, the testes were also thought to be the target of SARS-CoV-2. Whereas growing evidence for the presence of the viral particles in the testicular biopsies from patients infected with SARS-CoV-2 is highly limited. To date, several studies (10, 17, 37–40) reported testicular histology outcomes in COVID-19 patients. In these studies, testicular/epididymal pathological analysis were performed on deceased COVID-19 patients, and at least 5 testicular samples positive for SARS-CoV-2 particles were identified. However, these studies analyzing SARS-CoV-2 in testicular biopsies have based on deceased COVID-19 patients, which may be limit the explanation whether there were SARS-CoV-2 viral particles in predominantly mild COVID-19 patients. It is necessary to further study the pathological histology of testis in mild and moderate COVID-19 patients to determine whether SARS-CoV-2 can be detected in this population.

## The Feasibility of Sperm Cryopreservation

From the current point of view, the control of the COVID-19 epidemic will take a long time. At this stage, the fertility preservation center needs to update part of its working procedures to prevent the outbreak of COVID-19 from within (41). Moreover, in case of emergency, it is also necessary to face the fertility preservation demands of COVID-19 patients, who may be in the incubation phase, recovered phase, and even acute phase of critical illness. Although ART are being preferably cancelled or postponed during this pandemic, fertility preservation is an emergency requirement, as patients undergoing genotoxic treatments may induce transient or permanent sterility. Even if fertility preservation centers do not plan to cryopreserve sperm for COVID-19 patients, they will encounter people who need fertility preservation in high-risk environments. Therefore, cryobanks should make necessary preparations to ensure that they have the ability to cryopreserve sperm. At least professional consultation and advice should be formulated to meet the individual requirements of such patients.

The presence of virus in semen is not new to researchers, who have long known that semen may contain various viruses (42). During semen processing, laboratory operators are at high risk of transmission for the direct exposure to semen samples (43, 44). Sperm obtained from patients with viral illnesses, such as human immunodeficiency virus (HIV) infection and hepatitis, must be treated with special precautions to reduce exposure of the non-infected partner and cross-contamination of reproductive tissue within the laboratory (45). In practice, many laboratories have set up good safety protection systems and methods to lessen virus particles in semen. Although most studies have shown no detectable virus in ejaculate of COVID-19 patients, considering the special characteristics of SARS-CoV-2 transmission, we should not relax our vigilance, strengthening of precaution during semen handling procedures is still crucial.

Another serious concern is the potential cross-contamination during cryostorage stage, as most microorganisms can survive for a long time in the ultra-low temperature of LN_2_ (46). There has been controversy about the research results of virus cross-contamination in ART cryobanks. Bielanski and colleagues clearly showed an absence of cross-contamination from infected semen and embryo straws to non-infected samples stored in the same LN2 tanks; furthermore, they reported no virus contamination in embryos vitrified in sealed cryovials or straws (47, 48). Cobo and colleagues also failed to detect the presence of viral RNA or DNA sequences in LN_2_ used for oocyte or embryo vitrification in patients with seropositive for HIV, hepatitis C virus, and hepatitis B virus (49). For COVID-19-positive men, given that only very low titres of SARS-CoV-2 have been detected in non-respiratory sites, some studies consider the risk of significant virus shedding into semen is low (50). However, the possibility of cross-contamination between cryopreserved sperm samples during storage in LN2 is difficult to determine in the phase where this “low” risk is estimated merely (51). Now that most viruses are able to survive in LN_2_, contamination of other samples by virus invasion through flowing LN_2_ into broken or poorly closed cryovials/tubes is possible (52). Hence, cryobanks must be aware of the possible presence of SARS-CoV-2 in cryopreserved sperm and LN_2_, and take effective measures to minimize the aforementioned risk. If cryobanks plan to offer sperm cryopreservation for COVID-19 patients, some suggestions based on expert opinion informed by the literature should be followed.

**Figure 1:**
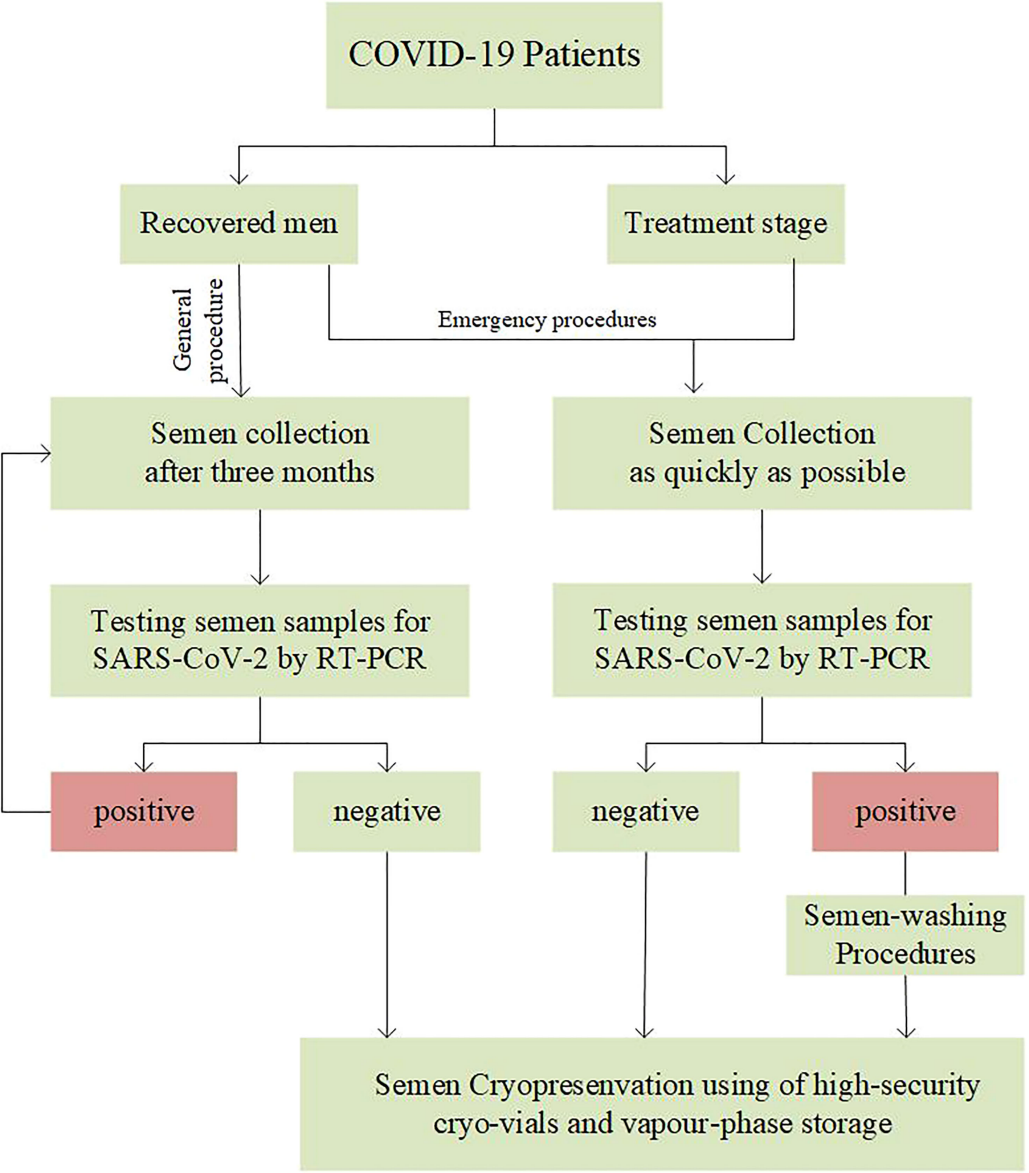
A decision path for sperm cryopreservation of COVID-19 patient.

- Managers of the cryobanks should be very prudent, and invite health authorities, including reproductive ethics committees, to evaluate their own conditions and facilities while providing regulatory standards (53).
- It is essential to establish the suitable precaution procedures and conduct strict training for the staff. If possible, dedicated areas should be set up to receive COVID-19 patients and collect samples (54, 55).
- Face-to-face interactions should be minimized with COVID-19 patients. Video conferencing, telephone and other online consultations can be used to collect the patient’s epidemiological history and assess the possible hidden risks (53). Meeting COVID-19 patients who want to fertility preservation, andrologists should give corresponding suggestions based on the decision path for sperm crypreservation of COVID-19 patients by managers of the cryobanks (**Figure 1**).
- For recovered patients, considering the persistence and half-life of SARS-CoV-2 in the body, it is recommended that sperm cryopreservation could be provided after 3 months in non-emergency situations (56). Especially, referred to patients with long COVID-19, 6-month interval or more should be suggested after the typical symptoms disappear.
- If recovered patients present with any suspected clinical of COVID-19 symptoms at cryopreservation stage, the cryobanks should initiate emergency procedures to diagnose whether they are COVID-19 recurrences, and discuss how to dispose of cryopreserved sperm. It should be noted that the sperm cryopreservation of patients with reinfection should be postponed.
- Urgently, such as COVID-19 inpatients with cancer who need fertility preservation, the cryobanks should invite the reproductive ethics committee to convene a meeting to fully evaluate the safety before starting the cryopreservation procedure.
- In andrology laboratories, safety cabinet class II t is recommended when handling semen of COVID-19 patients (57). One should take extra-care while dealing with semen. Once the semen is examined and handled, all single-use materials should be discarded in individual bins and disposed of immediately.
- In view of results of studies on SARS-CoV-2 in semen of COVID-19 patients have been controversial, SARS-CoV-2 testing of semen should be considered before cryopreservation. Based on 56 recommendations, RT-PCR assays was the index test more recommended for the diagnosis of SARS-CoV-2 (58).
- In the case of sperm cryopreservation, high-security cryo-vials should be used for all COVID-19 positive males. Cryo-vials should be stored independently with warnings labels.
- For unwashed semen samples or those awaiting viral test results, using of a separate, vapour-phase storage is recommended to minimize risk (59).
- Considering that SARS-CoV-2 may be present in other tissue, direct freezing sperm obtained by surgery should be avoided. Repeated washing and viral test procedures should be observed before cryopreservation (41).
- In the worst case of a positive semen obtained by patient with no further opportunity of sampling, sperm-washing procedures such as double-density gradient followed by swim-up can be used to dilute virus present before cryopreservation (60).
- Do not use COVID-19 positive males’ sperm until there is no evidence to prove the safety of these samples. When the cryopreserved sperm can be used in ART, the risk of transporting the samples between centers and the safety of the personnel working in the laboratory during thawing and handling should be considered (61).

## Discussion

Despite worldwide efforts to prevent and control the COVID-19 pandemic, SARS CoV-2 is still widespread in many countries and regions. As any emergent disease, numerous studies have been carried out to better understand characteristics of the virus and its short-and long-term repercussions on health status. So far, studies have strongly shown that SARS-CoV-2 can cause impairment of male fertility. The conclusion poses a distinctive problem to the cryobanks about how to carry out male fertility preservation during the pandemic. On the one hand, among so many COVID-19 patients, some do have the requirement of fertility preservation, otherwise they may never get their own offspring. Therefore, the health authorities should be fully aware of the fertility preservation demands of COVID-19 patients, and organize experts to issue the possibility of fertility preservation (62). Under the consensus formulated by experts and the suggestions recommended in the present article, the cryobanks could develop detailed preventive and operating procedures to carry out male fertility preservation for COVID-19 patients.

## Author Contributions

YW and XX conceived the review. YW, XZ, ZW wrote and reviewed the paper. All authors contributed to the article and approved the submitted version.

## Funding

This work was supported by research funds from the Provincial Natural Science Foundation, Guangxi Zhuang Autonomous Region, China (Grant No. 2018GXNSFAA050115), Key Research &Development Program of Jiangsu Province (No.BE2018713), Open Subject of Jiangsu Women and Children health Society (JSFY202005), National Population Commission Open subject (YJJC201802)

## Conflict of Interest

The authors declare that the research was conducted in the absence of any commercial or financial relationships that could be construed as a potential conflict of interest.

## Publisher’s Note

All claims expressed in this article are solely those of the authors and do not necessarily represent those of their affiliated organizations, or those of the publisher, the editors and the reviewers. Any product that may be evaluated in this article, or claim that may be made by its manufacturer, is not guaranteed or endorsed by the publisher.
